# Exploring the tensions of being and becoming a medical educator

**DOI:** 10.1186/s12909-017-0894-3

**Published:** 2017-03-23

**Authors:** Ahsan Sethi, Rola Ajjawi, Sean McAleer, Susie Schofield

**Affiliations:** 1 Institute of Health Professions Education & Research, Khyber Medical University, Hayatabad Phase 5, Peshawar, Pakistan; 2 Centre for Medical Education, University of Dundee, Dundee, UK; 3 Centre for Research in Assessment and Digital Learning, Deakin University, Geelong, Australia

## Abstract

**Background:**

Previous studies have identified tensions medical faculty encounter in their roles but not specifically those with a qualification in medical education. It is likely that those with postgraduate qualifications may face additional tensions (i.e., internal or external conflicts or concerns) from differentiation by others, greater responsibilities and translational work against the status quo. This study explores the complex and multi-faceted tensions of educators with qualifications in medical education at various stages in their career.

**Methods:**

The data described were collected in 2013–14 as part of a larger, three-phase mixed-methods research study employing a constructivist grounded theory analytic approach to understand identity formation among medical educators. The over-arching theoretical framework for the study was Communities of Practice. Thirty-six educators who had undertaken or were undertaking a postgraduate qualification in medical education took part in semi-structured interviews.

**Results:**

Participants expressed multiple tensions associated with both becoming and being a healthcare educator. Educational roles had to be juggled with clinical work, challenging their work-life balance. Medical education was regarded as having lower prestige, and therefore pay, than other healthcare career tracks. Medical education is a vast speciality, making it difficult as a generalist to keep up-to-date in all its areas. Interestingly, the graduates with extensive experience in education reported no fears, rather asserting that the qualification gave them job variety.

**Conclusion:**

This is the first detailed study exploring the tensions of educators with postgraduate qualifications in medical education. It complements and extends the findings of the previous studies by identifying tensions common as well as specific to active students and graduates. These tensions may lead to detachment, cynicism and a weak sense of identity among healthcare educators. Postgraduate programmes in medical education can help their students identify these tensions in becoming and develop coping strategies. Separate career routes, specific job descriptions and academic workload models for medical educators are recommended to further the professionalisation of medical education.

(Tensions, Fears, Healthcare Educators, Medical Education, Postgraduate Programmes, Identity, Career Choice, Faculty Development, Communities of Practice).

## Background

The growing expectations of the regulatory bodies for medical educators to have formal training for their educational role and increased accountability has resulted in the professionalisation of medical education [1–3]. We have seen an increase in the demand for postgraduate qualifications in medical education [4, 5]. Globally, we have seen an increase in the number of providers (and subsequently candidates in these programmes) from 7 to over 124 and in the United Kingdom (UK) from 2 to over 31 in the last two decades [5, 6]. These programmes have been found to enhance theoretical foundations in educational practices resulting in increased self-efficacy and engagement in various educational scholarship activities [2]. The attainment of a postgraduate qualification has been associated with developments in roles, leadership positions, and expanded responsibilities in medical education [7]. The graduates also report needing to translate their academic learning to their educational practices in the workplace, which may lead to particular challenges and tensions, as yet under-explored.

In 1999 Marks [8] reported that multiple demands on time and credence given to medical education were the main tensions for those pursuing a career in medical education. Ten years later Zibrowski et al., [9] also identified the time-related dilemma of faculty with an interest and involvement in educational scholarship. They found that fragmentation (lack of protected time to work on educational projects), prioritisation (juggling the various competing responsibilities of clinical, teaching, and administrative or leadership roles) and motivation (institutional values and colleagues’ reactions to their involvement in education) interfered with faculty members’ ability to achieve their goals.

Hu et al., [10] explored the sustainability of academic careers in medical education. They found that universities valued research output over high quality of teaching. Appointments and promotions were based on clinical experience rather than formal qualifications or scholarship in medical education. In addition, there was smaller economic capital in terms of remuneration and research funding for education compared to their clinical and research roles. As a result, their participants were adamant that maintaining a clinician’s status was essential. However, Hu et al., only interviewed early career professionals with experience and academic roles in education from Australian and New Zealand medical schools irrespective of whether they had a qualification in medical education.

The Academic Careers Sub-Committee of the UK Clinical Research Foundation [11] identified barriers towards pursuing academic careers by healthcare professionals such as limited information on entry routes and subsequent career pathways in academia, and lack of structured and funded posts upon specialization. Hauer and Papadakis [12] also showed tensions over lack of reward for excellence in teaching and other educational achievements in a system that appears to only value research productivity. Such lack of recognition for scholarship of teaching and frequent marginalization of clinicians engaged in an academic career has been reported by Kumar and colleagues [13] as well. Their participants perceived teaching as a ‘low-status occupation’ that only complements research and clinical practice. They also reported that professional training and progression in academic medicine is long, inflexible and financially unattractive. However, this study explored the journey of academic clinician-educators from one medical school within a ‘research-intensive’ university, that assumed a dichotomy between teaching and research, which is not that simple [14].

Previous studies have attempted to identify tensions medical faculty may encounter with regard to their educational role, but not specifically those with a qualification in medical education. It is likely that those with postgraduate qualification face several of the identified tensions (such as time and pressures with juggling multiple roles) but also are likely to face additional tensions such as through translational work or from differentiation by others. These tensions may lead to detachment, cynicism and a weakened sense of identity among those who are active within medical education. Therefore, it is important to recognise such tensions and foster the development of educational identities in view of the multiple roles most educators fulfil [15]. Understanding the tensions related to identity formation as medical educators should help improve their experiences and aid the professionalisation of medical education. This study explores in detail, the complex and multi-faceted tensions of educators with qualifications in medical education at various stages in their career from academic centres worldwide. The research questions are:

For those who have or are studying a postgraduate qualification in medical education:

- What are the tensions experienced in becoming and being a medical educator?
- How do these tensions differ between junior and more senior educators?

## Methods

### Study design

The data described were collected in 2013–14 as part of a larger, three-phase mixed-methods research study employing constructivist grounded theory [16] to understand in-depth the transformational changes and development of professional identities among medical educators following a postgraduate qualification in medical education. A constructivist grounded theory approach is considered useful for understanding the processes of professional practice and identity formation [16, 17]. It involves the construction of a theoretical explanation of a phenomenon based on gathering, synthesising, analysing and interpreting practical experiences of the participants [16]. Here we focus specifically on the tensions identified by the participants as they become medical educators.

With foundations in pragmatism [18], we aimed towards a fresh interpretation of the data rather than a complete or final interpretation [19], therefore any preconceived theoretical frameworks were not forced upon the data to find a fit. The category ‘tensions’ evolved from an in-vivo code during our data analysis and was used in the literature with reference to community of practice (CoP) theory, which views learning as a process of participation within legitimate work practices and spaces influencing identity formation [20, 21]. The community, and one’s relationship with it become part of one’s identity, which can cause tensions as individuals struggle to reconcile the personal with the social [22]. Here we refer to tensions as the internal/external conflicts or concerns experienced towards identity formation as medical educators during and after a postgraduate qualification in medical education. Key decisions made during the data collection, management and analysis were discussed among the researchers before subsequent action, providing researcher triangulation.

### Study setting

The study was conducted with students and graduates from the Centre for Medical Education (CME), University of Dundee. These qualifications are offered to a diverse range of national and international students from various health professions e.g., medicine, dentistry and nursing. According to CME records (Nov 2015), the total number of students and graduates worldwide was 2144 and 2992 respectively at that date.

### Data collection

Ethical approval was obtained from the Research Ethics Committee, University of Dundee. The first author (AS) was a PhD student and tutor on the Dundee programme at the time of the study, potentially putting him in a position of power in relation to the students. To minimise this risk, the first author was not a part of the assessment team for that cohort of students. Invitation emails with consent forms and participant information were sent by the course’s office to the programme’s students (at the time of the study) and by the first author to the graduates.

The participants were graduates of the programme between 2008 and 2012, who had agreed to participate in this qualitative study (*n* = 129) during our previous survey study [2] and the full-time face-to-face students (*n* = 14) in the 2013/14 session in Dundee. The sample size for the graduates was not predetermined; instead an iterative approach of data collection and analysis was undertaken until sufficiency of explanation was judged to be reached [23] in line with constructivist grounded theory [17, 24]. An iterative process involves going back and forth between collection and analysis with one informing the other. Such process helps ensure the comprehensiveness and diversity of responses. The decision to stop the data collection was made by the research team based on judgements of the preliminary analysis.

The experiences of the participants were explored through semi-structured interviews conducted by AS. The participants were asked about their fears of and tensions around becoming and being a healthcare educator. Participants were interviewed using landline, Skype and face-to-face meetings depending upon individual circumstances and their feasibility. The interviews from landline were recorded through a RETELL 156 Telephone Recording Connector device with input into a stereo recorder. Skype interviews were recorded using MP3 Skype recorder 4.2. All interviews were audio-recorded and transcribed, and transcripts were checked for accuracy against the recordings by the first author. The remaining authors are academic medical educators and were all involved in the Dundee programme at the time of the study. They were not involved in any of the recruitment or data collection and the data were de-identified before data analysis.

### Data analysis

The data were imported into qualitative data management software ATLAS.ti 7. Analysis was informed by a constructivist grounded theory approach [16, 25]. Three transcripts each from the students and the graduates were initially coded by the researchers independently. Any differences in interpretation were reviewed and either new themes created or existing themes collapsed through negotiation. The codes were identified through active interaction with the data and reading each line, incident and segment to construct codes for fit and relevance. The initial codes were examined and synthesised into a number of conceptually related themes. The analytical process involved constant comparison of data with data, data with themes, themes with themes, and themes with concepts, across all the participants for commonalities, differences and recurring patterns. The coding framework and relationships between themes were developed through regular team meetings, discussion and negotiation by the research team to make sense of the whole. CoP was a lens through which the data were interrogated.

## Results

### Participant demographics

Participants (*n* = 36) were at varying stages in their professional careers, from different countries, and with a diverse range of characteristics (Table 1). The professional background was predominantly medicine. The face-to-face students (*n* = 9) were all international students, whereas half of the graduates (*n* = 27) were UK nationals at various stages of their career. Many of the graduates had taken the course as a distance learner, with study time ranging two to nine years, indicating long exposure to medical education for some participants. Educational roles included deans, head of departments, directors, professors, assistant professors, consultants, project leaders, lecturers, clinical registrars and PhD students. Two face-to-face students were between jobs, and over the period of the course they actively started looking for employment as well as PhD opportunities in medical education outwith their home country.

**Table 1:** Characteristics of interview participants

Characteristics		Frequency	
		Students	Graduates
Gender	Male	2	12
	Female	7	15
Age	Under 30 Yrs.	3	1
	30–45 Yrs.	6	13
	Over 45 Yrs.		13
Qualification	Certificate	1^a^	9
	Diploma	2	2^a^
	Masters	6	16
Year of Graduation	2008	N/A	11
	2009		2
	2010		4
	2011		3
	2012		7
Profession	Medicine	8	22
	Dentistry	1	1
	Med Ed		2
	Nursing		1
	Pharmacy		1
Nationality	United Kingdom		12
	Thailand	4	2
	Australia	1	2
	Pakistan	1	1
	Saudi Arabia	1	1
	United States		2
	Canada		2
	Egypt	1	
	Indonesia	1	
	South Africa		1
	Sri Lanka		1
	Chile		1
	Denmark		1
	Nepal		1

^a^currently enrolled for Diploma/Masters

### Tensions of being and becoming a medical educator

Through analysis of the transcripts, we identified six tensions associated with both becoming and being a medical educator (Table 2). These tensions are not mutually exclusive and do not occur in isolation.

**Table 2:** Tensions as a medical educator

Tensions common to both active students and graduates
Balancing multiple intersecting identities
Educator careers are undervalued
Educator careers are less well defined
Translating theory to practice
Educator careers are financially disadvantaged
Challenges towards further development and staying up to dateᅟ ᅟᅟ

#### Balancing multiple intersecting identities

Students and graduates expressed tensions with finding the right balance between education and clinical and were worried about their loss of identities if they choose to be full-time educators. They expressed a concern that choosing one identity would result in losing the other. The clinical society might disregard them as educators and they might not be taken seriously in the medical world. A Masters’ student from Indonesia mentioned:

*‘If I become a full time educator what will happen with my clinical career that’s one of my worry also. I do not really know after this it’s like do I really want to sacrifice my clinical career now and become full time educator and I still can’t answer that question really well now’* (Male-Medicine-Masters-Student#4)

The participants with a clinical background mentioned having to juggle multiple roles including clinical and educational roles. With their new educational responsibilities, they found it hard to maintain a work-life balance. A foundation programme tutor and associate medical director reported:

*‘The time it takes to do it properly, I worry that it could impact on my patients and … the impact that it has on my home life as well … so those tension between my clinical work but also my home, my work life balance’* (Female–Medicine-Certificate-Graduate#168)

#### Educator careers are undervalued

Participants reported a negative bias and less regard towards those wishing to pursue a career in education compared with clinical specialities. Education was seen as less valued in comparison with other career tracks of a health professional in their countries. Despite the associated career progress, their colleagues questioned its need and importance. A clinical skills tutor mentioned:

*‘Education [is] perhaps less respected than the clinical disciplines … when I talk to people who are clinicians … they ask what are you doing and I say education and they are kind of like oh you know I am wondering if that is a real speciality … and sometimes you kind of get sick of explaining that to people it might be easier to just say oh I am a surgeon ((laugh))’* (Female–Medicine-Diploma-Student#1)

Others reported active marginalisation of those working in the medical education department:

*‘People who didn’t cheer in the curriculum reforms so they hate the name medical education … people who forced us to change our curriculum who forced us to integrate and teach less so they hate the medical education department’* (Female–Medicine-Masters-Student#3)

#### Educator careers are less well defined

Both students and graduates also stated that an educator career track was not defined in their institutions. Job descriptions and promotion policies were not clear for educators, and they were judged on the same criteria as clinicians or researchers. They expressed a concern that, being an emerging speciality, people did not have much idea of how medical educators impacted on healthcare. A continuing education director from North America stated:

*‘So my job description is as a clinician educator which is probably the least well defined of all the job descriptions in terms of what the expectations are and what the criteria are for promotions.’* (Male–Medicine-Masters-Graduate#94)

#### Translating theory to practice

Participants also found it challenging to enact their learning due to various factors involved in the process beyond their control. They expressed tensions when translating knowledge learned during the programme to the specific-contexts of their workplaces as represented by the following excerpt from a lecturer of medical education:

*‘Unfortunately you cannot impose any programme on professionalism from another country … because professionalism is a culture specific issue, what is professional for you in Pakistan, may not be professional for me in Egypt or in UK … even the way of dressing of a doctor makes it different … so you need to tailor your own programme on professionalism which is a challenge’* (Male-MedEd-Masters-Graduate#30)

This theory-practice gap is also highlighted in the following excerpt of consultant neurosurgeon and clinical teacher from Thailand:

*‘In the accident and emergency unit that for me it is difficult to do it [feedback] readily because I had to practice at the same time … I had read about feedback that … needs to be done immediately but for me it’s very difficult in real practice’* (Female-Medicine-Diploma-Graduate#202)

#### Educator careers are financially disadvantaged

The participants also mentioned that pursuing a career focussed on medical education placed them in a weaker financial position in comparison to their clinical colleagues. Moreover, funding for educational projects was less than in the biological and medical sciences, making it almost impossible to receive equal funding for their institution. Yet they were being judged based on the same metrics as those in the biological and medical sciences. A head of medical education centre explained:

*‘The financial issue for example in Sri Lanka majority of doctors do private practice and they earn a lot in my case I depend highly on the salary I receive from the university … there are tensions about whether I took the right decision because I am at a relatively financially disadvantaged position’* (Male-Medicine-Masters-Graduate#59)

#### Challenges towards further development and staying up to date

Participants were concerned about their further development in medical education. They mentioned various reasons such as unavailability of PhD programmes, lack of mentors and inaccessibility of the literature in medical education locally. An MD from Saudi Arabia mentioned:

*‘I say now that I want to do a PhD but I might not get to … there aren’t many graduate programmes in medical education around the world it’s like 20 programmes in PhD so what if something does not come up then you would have to consider other avenues that would take you away from education’* (Female–Medicine-Masters-Student#2)

The graduates were challenged with further development as they came to recognise that medical education was a vast speciality with sub-specialities in teaching, learning, assessment and curriculum. Therefore, it had become difficult for them as a generalist to keep up-to-date in all areas of medical education. One senior clinical teacher from the UK reported:

*‘You like a little bit of everything so I am not going to be a specialist in assessment. In terms of the education, I like to dabble in all sorts, so trying to find the balance to keep up-to-date with all is interesting’* (Male-Medicine-Masters-Graduate#34)

Graduates also expressed a concern that they were expected to know everything about education in their institutions. They were at times asked to take on responsibilities that were not relevant to their interests or competencies. A director in Continuing Education from Canada stated:

*‘I think people end up assuming that education is just one thing and that someone who has an interest or training in education can do everything related to any aspect of education, so … my thesis was in postgraduate education but the responsibility I was given when I started here was more in a realm of continuing education.’* (Male-Medicine-Masters-Graduate#94)

### No fears

Interestingly, most of the graduates with extensive experience in education and 45+ years of age (more senior educators) reported no fears, rather asserting that the qualification gave them job variety. In the words of a clinical tutor from UK:

*‘I do not think I have any fears I think it gives me lots of options I think it gives me variety at my job because … I facilitate research … I do a lot of clinical work and about a third of my job plan is involved in teaching now, so it’s a nice mixture.’* (Female-Medicine-Masters-Graduate#72)

## Discussion

The participants reported multiple fears and tensions associated with becoming and being a healthcare educator. The tensions commonly identified by the graduates and students e.g., being unable to balance various roles, lack of regard for educators, and being financially disadvantaged are in line with those identified by healthcare educators in other studies [8–13]. Our analysis identified some additional tensions for those pursuing or having a qualification in medical education such as managing intersecting identities, challenges towards further development, keeping up-to-date with new research and unrealistic expectations from colleagues to know everything about medical education. Interestingly, the more experienced senior educators (45+ years of age) reported no tensions or fears. Many of these tensions related to becoming and being an educator are magnified by the disciplinary and organisational culture and policies in the workplace. Therefore, the participants’ fears were influenced by contextual factors and their response to these factors was determined by personal factors. CoP theory helps explain why there may be tensions between the personal and social, as individuals construct their identities and endeavour to find a place in the community [22]. The graduates extensively used ‘you know’ while describing their fears. This suggested that these fears were not specific to these individuals but commonly known and shared experience of being a healthcare educator.

The participants found it difficult to balance their educational roles or pursue scholarly activities with other clinical responsibilities and administrative loads, as also reported by Edwards [26]. They reported multiple time demands with no protected time for carrying out scholarly activities. They often sacrificed personal time, thus affecting their personal lives. Zibrowski et al., [9] also reported similar constraints on the faculty with interest and involvement in educational scholarship. Such time related constraints with little time to improve teaching, assessment or curriculum makes the development of further competencies in education challenging for the educators [27].

Having an active clinical practice was seen as important and fundamental in supporting medical education roles. The process of professional identity formation meant that students were working out how to balance their clinical and educational identities with some considering becoming fulltime academics in medical education. They saw themselves at the crossroads between clinical and developing educational identity, and wondering which direction to take. This tension arises from a comparison of their internal identities with their possible selves i.e., becoming an educator [28]. In cases of conflict, this negotiation may result in rejection of the professional roles (new or old identities) or other acts of resistance such as inappropriate behaviours [29, 30]. The Early Careers Working Group at the Academy of Medical Educators also reported this career identity crises [31]. This situation probably arises because medical education is still in its nascent stage, and may resolve with the increasing professionalisation of the speciality, although many entering medical education as health professionals will have to make choices about managing their clinical and educational roles and identities.

Brownell and Tanner [32] found that many universities offer no rewards for excellence in teaching or introducing evidence-based strategies. The participants in this study reported a similar lack of appreciation for educational roles in their institutions. In the literature, many such challenges are common to teaching in clinical environments that value clinical practice and research productivity [12, 13, 33]. Kumar and colleagues [13] also reported that teaching is perceived as a ‘low-status occupation’ and only useful as an add-on to research and clinical practice. This further compounds the perceived need to juggle multiple roles and of managing multiple intersecting identities within a broader community that does not necessarily value their educational expertise. Despite improved self-efficacy and development as leaders, faculty who feel their contributions are unrecognised are more likely to burn out and are less likely to continue contributing [13, 34].

Participants felt that medical education, being a relatively new field of practice, was poorly defined as a career. The Academic Careers Sub-Committee of the UK Clinical Research Foundation [11] also pointed out this limited information on entry routes and subsequent career pathways in academia, and lack of structured and funded posts for educators. Sethi [7] reported various extrinsic motivations for these participants towards enrolment. For example, some enrolled because they perceived the qualification would bring positive career prospects, while others were recommended and sponsored by their home institutions. This suggests an increased differentiation of the roles of medical educators and a need for qualified educators. It also shows a trend towards recognition of medical education as a speciality. This shift in focus and investment towards development of medical education at the level of policy makers and leadership shows that there is a promising future for early career medical educators pursuing it full-time. As highlighted in the Table 1 above, the increased proportion of under 30 years old students in 2013/14 cohort in comparison with the graduates between 2008 and 2012 suggests younger healthcare professionals are also recognising medical education as an area worth specialising in. This is a promising positive shift for the valuing of medical education that might not be shared by all clinical colleagues in the workplace.

The participants also resented that the promotion policies in their institutions are not clear for educationalists, and they are often judged using the same criteria as clinicians or researchers in their institutions (e.g., amount of awarded grant funds). This resentment towards promotion assessment against a metric that did not relate to teaching quality was also reported by early career medical educators in Australian and New Zealand medical schools [10] and clinicians engaged in an academic careers [12, 13], suggesting a worldwide phenomenon.

As with any new field of scholarly activity medical education faces multiple challenges such as low levels of funding, lack of recognition and reduced opportunities for career development [10, 13, 15, 35]. The participants mentioned that their efforts are undervalued and under-supported financially in comparison with those working in other areas. Moreover, they were concerned that a full-time career in medical education might be personally financially disadvantageous, as academic salaries rarely match those of clinicians. Goldszmidt et al., [36] reported that faculty do not receive financial support for their work and they are discouraged from participating in educational conferences because of the associated costs. It has been called a ‘Cinderella discipline’ [10]. Despite all these concerns, the graduates continued to invest in education owing to their interest and passion. Most of them struggled but continued to work out-of-hours, affecting personal and family life. This is associated with altruistic behaviour, which is a common trait among those classifying themselves as members of a profession [37]. However, society’s acceptance of health professionals with full-time careers in medical education will be critical to their retention and success [9].

Participants recognised the complexity of practice and highlighted the gap between theory and practice. They were also concerned about the contextual nature of practice in medical education and the translational work needed to effect practice change in the workplace. Effecting educational change requires strong educational leadership and sense of self in the face of resistance and under-valuing. The flipside is that the interplay of these tensions may lead to detachment, cynicism and a weak sense of identity among healthcare educators leading to decreased ability or motivation to effect changes in the workplace and to seek further professional development.

Participants reported concerns about their further development as medical educators in their home countries after graduation. The lack of available further training in medical education conflicts with other mature specialties such as basic and clinical sciences in which the training progression is clearly defined and well-trodden [11]. Graduates also reported more practical concerns regarding staying up-to-date and being seen as an expert in medical education having returned to their institutions with a qualification. Staying current with the literature in medical education is an important competency of expert teachers [38]. Faux [39] estimated that for each working day, it would take 75 min for a physician to keep themselves up-to-date with the medical education literature, which is not practical given current workload demands in the healthcare sector. All such concerns highlight a need towards continued professional development programmes as well as developing sub-speciality courses and for preparing specialists with advanced knowledge and skills in specific areas of medical education such as assessment, curriculum development, educational research methodology and technology-enabled learning. Specialised Masters’ programmes or postgraduate programmes with optional specialist modules are already being offered in topics such as simulation. However, such specialisation requires accepted career paths and positions within the academia and healthcare settings.

Interestingly, all the graduates who were more senior (45+ years of age) reported no anxiety as educators. In fact, they reported that the qualification gave them job variety, which was valuable to them. However, mention of this ‘variety’ may evidence their view of medical education being a routine professional activity rather than a career in its own right [10, 40]. Alternatively, it may highlight that the senior educators were secure in their career choices and intersecting identities within their communities and had managed to find ways of managing competing demands on their time.

### Limitations

The findings were based on participant self-reporting and not correlated with observed behaviours. Also, the study was based on participants from one programme but from many countries and work institutions. Yin [41] argues that case study research closely examines the data (micro-level) within a specific context and provides detailed insights into the behaviours when little information is available regarding a contemporary phenomenon. We have assumed that those who graduated in 2008 have more educational experience than those who graduated in 2012 and that those over 45 years of age are more senior educators than those under 30 years.

Thirty of thirty-six participants are medical doctors. Two participants (30–45 years age group) who mentioned medical education as their profession (Table 1) were also doctors but did the qualification early in their professional career. The graduates belonging to professions other than medicine in our study were all above 45 years of age and reported having no fears, as was found with this age group from medicine. The findings may be transferable to healthcare educators broadly as a diverse range of professionals are teaching in the medical schools [15] and many of these tensions are related to the policies and practices in the workplace. However, further study is needed in this area, as to assume these findings are transferrable to other healthcare educators on the current data would be premature. We have reported participants’ year of graduation along with a description of their roles, acknowledging we could have asked more questions relating to their training. However, we were keen to keep the interview guide short to maximise engagement. Despite these limitations, the study achieved its aims; it complements and extends the findings of the previous studies.

### Implications of the study

Kumar and colleagues [13] found that beliefs and values related to teaching have a strong impact on identity formation of healthcare educators. Therefore, understanding these tensions are critical to successful academic careers [8, 42]. Postgraduate programmes in medical education could help their students identify these tensions and develop coping strategies. These programmes should also equip the graduates with skills around educational change management and knowledge translation that takes account of the context. Postgraduate alumni mentoring programmes may further assist becoming medical educators in navigating the multiples tensions and poorly defined career paths highlighted in this research.

Health professionals usually represent and belong to clinical disciplines from which they primarily derive their knowledge. However, when they begin to take on an educational role, they should be judged against the stated criteria for an educator in planning, policy, delivery or research rather than their clinical specialty. Without this the educators will be under pressure to spend the majority of their time in clinical practice or research rather than developing and delivering high quality of teaching or innovations in practice. Medical educators represent a heterogeneous workforce with varying responsibilities for curriculum design, assessment, research or management [43]. Therefore, it is difficult to encompass their roles and competencies within a set of standards. The descriptors for different categories of the UK Professional Standards Framework [44] and Academy of Medical Educators [45], may be able to assist in professionally validating the capabilities of medical educators.

Medical educators should be given adequate opportunities, including protected time and funding in order to improve their teaching, introduce innovations and interact with an educational community in the workplace. Separate career routes, specific job descriptions and academic workload models for medical educators are recommended to further the professionalisation of medical education and benefit the educators, and in turn students and patients in the long term [37, 46]. We see this specialisation also occurring through for example the Faculty of Surgical Trainers, an initiative of The Royal College of Surgeons of Edinburgh, or the Academy of Medical Educators’ recognition of specific education competencies. Further in the UK the Teaching Excellence Framework (TEF) is currently being developed to create a dynamic and internationally competitive teaching sector, and also to encourage the professionalisation of educational roles in HE [47]. This framework mirrors the Research Excellence Framework [48] which is used for assessing research and allocation of governmental funding to UK higher education institutions. The impact of the REF on improving the quality of research has been reported recently [49]. However, teaching and learning is complex, multi-faceted and dynamic, thus identifying TEF assessment metrics which encourage transformation of teaching will be a challenge.

With medical education becoming a full-time career, there is a growing demand for doctoral programmes in medical education and robust research studies. However, these programmes are currently concentrated in the North America, Europe, Africa, Australia and New Zealand [50]. Hence, there is a need for more doctoral programmes with equitable distribution around the world. An infrastructure with accessibility to medical education literature and a critical number of individuals having a Masters/PhDs in medical education is a pre-requisite, otherwise, the quality of research in medical education will be suboptimal. To overcome the shortage of PhD supervisors, Riesenberg et al., [27] recommended hiring non-physician professionals with expertise in education as they can offer added value through their alternative perspectives on problems in medical education. Tekian [50] also recommended considering other options leading to specialisation in medical education, such as doctoral training in other disciplines, collaborating with those in medical education to foster disciplinary diversity and advancement in medical education research.

## Conclusion

This study has highlighted tensions specific to being and becoming medical educators such as managing multiple roles, lack of PhD opportunities, the challenge of staying up-to-date with new research and unrealistic expectations from colleagues to know everything in medical education. A comparison of tensions expressed by participants at various stages in their career indicates that medical educators move through different stages of emotional experience as they find their place, and develop a sense of belonging. Addressing the marginalisation of medical educators within their disciplinary and organisational cultures, and assisting becoming educators in navigating their careers through tailored workload and promotion policies, access to further educational opportunities, knowledge about possible tensions and access to mentoring would seem priorities.
